# Primary hyperparathyroidism presenting as a brown tumor with hypercalcemia crisis in a second-trimester pregnant woman: A case report

**DOI:** 10.1097/MD.0000000000025968

**Published:** 2021-05-21

**Authors:** Yun Xu, Yingying Yu

**Affiliations:** a International Medicine Department; b The Second Affiliated Hospital Zhejiang University School of Medicine, Hangzhou, China.

**Keywords:** brown tumor, hypercalcemia, parathyroid adenoma, primary hyperparathyroidism, second-trimester

## Abstract

**Introduction:**

Primary hyperparathyroidism (PHPT) in pregnancy is rare and unrecognized because the maternal physiological adaptations blurs the symptoms. There is no standard treatment strategy for maternal PHPT. Early diagnosis and interventions can prevent catastrophic consequences to the mother and fetus.

**Patient concerns:**

A 31-year-old Chinese woman was admitted, due to a lump on the left lower leg for 4 months. The patient complained of mild pain in the left lower leg following exercise that could be relieved after a short rest. The patient was at 18 weeks of gestation, and the growth of the fetus was normal. The patient has a 3-year history of hypercalcemia and a 2-year history of nephrolithiasis. No family history of hypercalcemia and endocrine tumors were present.

**Diagnosis:**

Laboratory tests demonstrated high serum calcium level of 3.84 mmol/L, parathyroid hormone 1393 pg/mL, alkaline phosphatase 488 μ/L. Ultrasound showed a 22.4 mm × 7.8 mm solid nodule in the left lower lobe of the thyroid gland. Based on these findings, the patient was diagnosed with PHPT.

**Interventions:**

The patient accepted continuous renal replacement to reduce ironized calcium level. Parathyroidectomy was performed at the 19th week of gestation. Threatened abortion occurred 2 days after the surgery, and magnesium sulfate was used to prevent the abortion. Calcium gluconate, calcium carbonate and vitamin D3 were used to treat the hypocalcemia that occurred 5 days after the surgery.

**Outcomes:**

Pathology examination demonstrated the parathyroid adenoma. Abortion was prevented using magnesium sulfate and hypocalcemia was cured with calcium gluconate, calcium carbonate and vitamin D3. At 38-week of gestation, the patient (ionized calcium level: 2.16 mmol/L) delivered a healthy female baby weighing 2700 g with 10/10 Apgar. Till now, both the mother and infant showed no complications.

**Conclusion:**

Maternal PHPT is rare and challenging to diagnose, causing life-threatening complications to mother and fetus. Any decision regarding surgery for a pregnant woman with primary hyperparathyroidism is more complex than in men or nonpregnant women. The decision should be made based on the severity of hypercalcemia and symptoms.

## 1 Introduction

Primary hyperparathyroidism (PHPT) is an endocrine disorder characterized by increased plasma parathyroid hormones and calcium, and usually occurs as the result of sporadic parathyroid adenomas or carcinomas. It is the third most frequent endocrine disorder, following diabetes mellitus and thyroid disease. PHPT is defined as the persistent elevation of serum calcium level in response to elevated or abnormal PTH levels.^[1]^ A previous study showed that PHPT affects 0.3% of the general population but twice higher in women,^[2]^ and the situation is similar in pregnant women and nonpregnant women.

PHPT during pregnancy causes life-threatening complications for mother and fetus. The maternal complications include nephrolithiasis, pancreatitis, muscle weakness, hyperemesis gravidarum, preeclampsia, and hypercalcemic crisis. The fetal complications include intrauterine growth retardation, permanent hypoparathyroidism, prematurity and intrauterine fetal loss.^[1,3–6]^ However, PHPT is challenging to diagnose because the symptoms could be hidden by maternal physiological adaptation. The increased maternal calcium can be transported to the fetus through the placenta, and the increased glomerular filtration rate also contributes to blunt the calcium level in the mother. The lack of characterized symptoms of PHPT during pregnancy is another reason for the misdiagnosis. A previous study of women with severe symptomatic hypercalcemia in pregnancy showed a significantly higher risk of developing maternal-fetus complications.^[4]^ Deteriorating hypercalcemia may cause a hypercalcemic crisis during pregnancy, a life-threatening complication to both mother and fetus. Therefore, early diagnosis and treatment of PHPT are critical. A case of pregnant women presenting as a brown tumor with hypercalcemia crisis due to parathyroid adenoma is reported in this paper.

## 2 Case presentation

**Figure 1:**
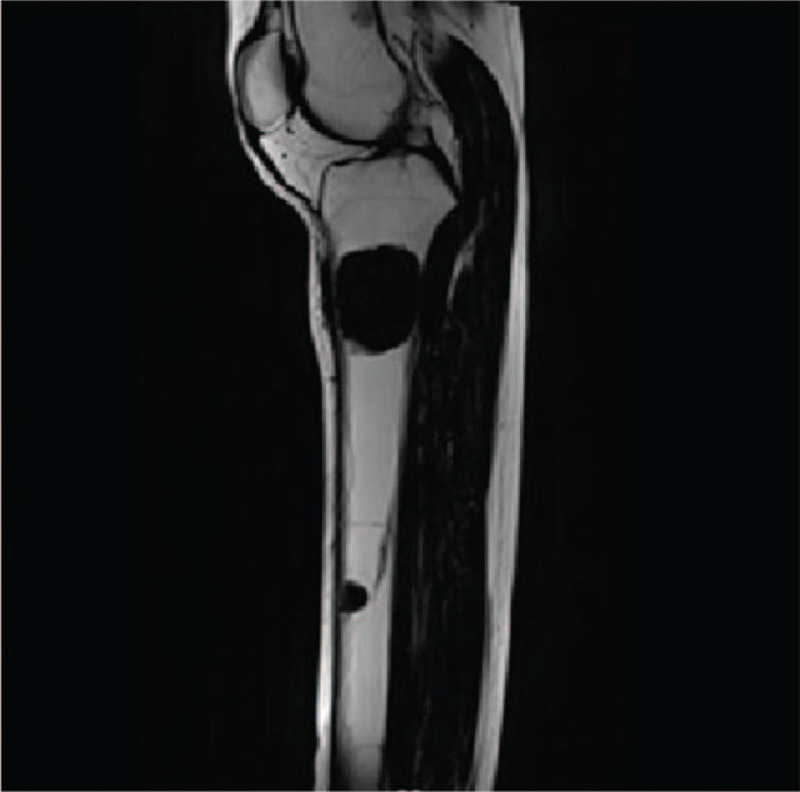
MRI of the patient showed a soft/hard tissue mass (37 mm × 30 mm × 42 mm) in the anterior/posterior compartment of the left lower leg.

**Figure 2:**
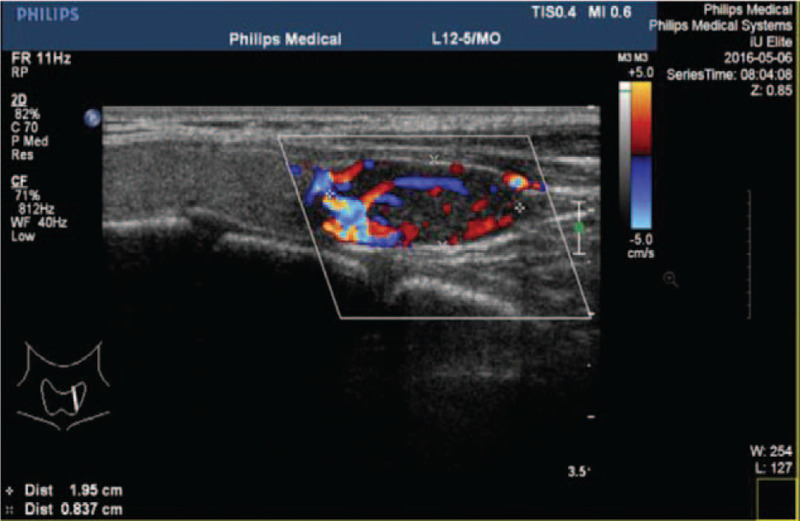
Ultrasound of the patient showed a 22.4 mm × 7.8 mm solid single nodule localized at the left lower lope of thyroid gland.

A 31-year-old Chinese woman was admitted due to a lump on the left lower leg existing for 4 months. The patient complained of mild pain in the left lower leg after exercise that could be relieved by short rest. The patient was 18 weeks pregnant, and the fetus was in normal condition. The patient had a history of hypercalcemia for 3 years and nephrolithiasis for more than 2 years without a family history of hypercalcemia or endocrine tumors. On physical examination, a deep-seated round mass was detected in the left lower leg of size 20 mm × 30 mm, showing no mobility and no pain. The laboratory tests demonstrated high serum calcium level of 3.84 mmol/L (reference range: 2.20–2.65), parathyroid hormone 1393 pg/mL (reference range: 15–65), alkaline phosphatase 488 μ/L (reference range: 30–120), hemoglobin 86 g/L (reference range: 113–151). The 24-hour urine calcium was in the normal range. MRI showed a soft/hard tissue mass (37 mm × 30 mm × 42 mm) in the anterior/posterior compartment of the left lower leg (Fig. 1). Ultrasound showed a 22.4 mm × 7.8 mm solid single nodule localized at the left lower lobe of the thyroid gland (Fig. 2).

### 2.1 Treatment

After a multidisciplinary consult, including endocrinology, maternal-fetal medicine, anesthetic and general surgery specialists, eucalcemic diet and aggressive hydration treatment were given to prevent acute pancreatitis before surgery. After 2 days of treatment, the patient complained of chest tightness and shortness of breath. The ionized calcium level was 4.2 mmol/L. The hypercalcemic crisis was diagnosed based on the ionized calcium level, and the patient was transferred to the intensive care unit to receive continuous renal replacement therapy (CRRT) for improving the calcium level. After 2 days of CRRT, the ionized calcium was back to 3.48 mmol/L and no more chest tightness was experienced. Parathyroidectomy was performed for the removal of the tumor at the 19th week of pregnancy. A single left inferior parathyroid adenoma with 20 mm × 7.8 mm in size was completely removed. The pathological examination showed parathyroid adenoma. One hour after the surgery, the ionized calcium level dropped down to 3.31 mmol/L and parathyroid hormone was back to 133.8 pg/mL. The patient's calcium and PTH levels were back to normal within 24 hours after the surgery. However, hypocalcemia occurred 5 days after surgery. Calcium gluconate, calcium carbonate and vitamin D3 were used to treat the hypocalcemia.

**Figure 3:**
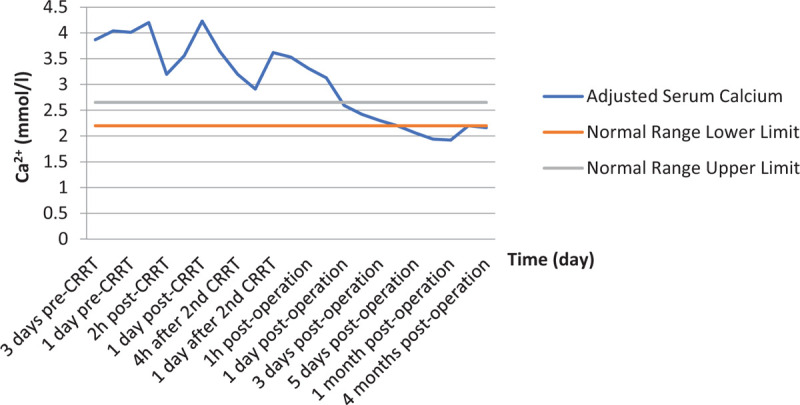
Summary of adjusted serum calcium. The results are from preoperative duration and 4 months after surgery. Normal range is given for reference (2.20–2.65 mmol/L).

**Figure 4:**
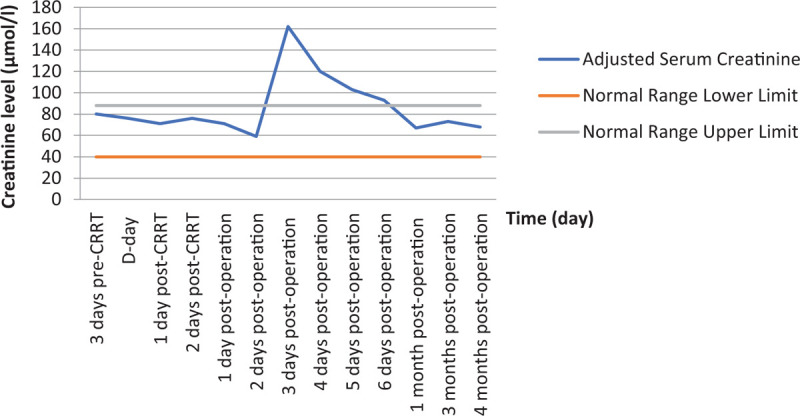
Summary of adjusted serum creatinine. The results are from preoperative duration and 4 months after surgery. Normal range is given for reference (40–88 μmol/L).

On the 3rd day after the surgery, the patient complained of lower abdominal pain with decreased fetal movement, without colporrhagia, and the laboratory test showed a low level of hemoglobin 62 g/L and high creatinine level 162 μmol/L (reference range: 40–88). Obstetric consultation considered that the pain was due to threatened abortion, and prescribed magnesium sulfate solution to prevent the potential abortion. The pain was relieved 1 day after treating with magnesium sulfate, and the fetal movement was back to normal. Then, blood transfusions with 2 units of suspension red blood cell were given from the 3rd day (1 time per day) after the surgery till the 5th day after the surgery to treat the anemia. Meanwhile, aggressive hydration was started for correcting the high creatinine. Retesting showed that the hemoglobin level was elevated to 78 g/L and the creatinine level was down to 103 μmol/L after 3 days of the treatment. The patient was discharged 6 days after the surgery with ionized calcium of 1.94 mmol/L, creatinine of 93 μmol/L. The details of calcium and creatinine levels are shown in Figures 3 and 4.

### 2.2 Follow-ups

At 38-week of pregnancy, the patient (ionized calcium 2.16 mmol/L) delivered a healthy female infant weighing 2700 g. The Apgar scores were evaluated at 1 and 5 minutes after birth, which were 10/10. Normal calcium levels for both mother and infant were confirmed in the postpartum period. The patient was discharged 3 days after delivery in a satisfactory and stable condition. The patient maintains serum PTH and calcium within the normal range during follow-ups. The female offspring is healthy without any complications during follow-ups.

## 3 Discussion

### 3.1 Diagnosis

Seventy percent to 80% of PHPT patients with hypercalceium are asymptomatic and are usually diagnosed incidentally,^[7]^ as manifestations of PHPT may be mixed up with common pregnancy-related complications. Early diagnosis is even more challenging in pregnant patients, because the calcium metabolism is changed due to the fetus’ needs for calcium, and calcium levels are not routinely measured during pregnancy. It is an uncommon clinical disorder in women of childbearing age. The prevalence of PHPT during pregnancy is unknown and may occur in less than 1% of cases.^[8]^ A diagnosis of PHPT in pregnancy should consider an elevated ionized calcium level and an elevated serum PTH level in the absence of other causes of hypercalcemia. Besides the laboratory tests, past medical history like renal stone, spontaneous abortion should be collected for the basis of diagnosis. In this case, the patient had a two-year history of renal stone and a three-year history of hypercalcemia. A comprehensive history would be helpful for diagnosis. Meanwhile, it is necessary to rule out familial hypocalciuric hypercalcemia and hereditary syndromes such as multiple endocrine neoplasia syndrome (MEN-1 or MEN-2), familial parathyroid hyperplasia syndromes or familial hypocalciuric hypercalcemia by testing 24-hour urinary calcium and creatinine levels.^[9]^ Common techniques such as computerized tomography and 99mTc-sestamibi scintigraphy to detect parathyroid adenomas or hyperplasias are not recommended in pregnant women due to the fetal risk of ionizing radiation. Therefore, neck ultrasonography is the current first-line choice for diagnosing parathyroid diseases in pregnant women.

### 3.2 Monitoring the level of calcium

Previous case reports showed that maternal and fetal complications could not be predicted based on duration or severity of hypercalcemia,^[5,10]^ Hirsch et al performed a retrospective cohort research to reevaluate the data with regard to the miscarriage risk associated with mild maternal hypercalcemia. The study of 74 pregnant women with a mean serum calcium concentration of 2.72 mmol/L showed no increase in spontaneous abortions relative to control participants without PHPT.^[11]^ Another retrospective cohort study of 1057 Danish pregnant women with PHPT compared with matched control women showed no difference in the rate of abortions.^[12]^ While Norman^[6]^ and McMullen^[10]^ showed that the risk of miscarriage in mothers with PHPT increased compared to the general population, even with low maternal serum calcium concentrations. If the fetus is exposed to maternal hypercalcemia, the fetal parathyroid function and development would be inhibited. Consequently, the fetus or infant would be at risk of neonatal hypocalcemia, neonatal tetany, and permanent hypoparathyroidism. A previous study reported that 2.85 mmol/L is a cutoff point for indicating the high risk for maternal and fetal complications.^[13]^ For those pregnant patients whose calcium levels were higher than 2.85 mmol/l, almost 50% of them lost their babies at the 12th week of pregnancy, and about 12% of infants suffered from hypoparathyroidism.^[6]^ When pregnant women have high serum calcium while being asymptomatic and untreated, maternal complications including nephrolithiasis, radiographic bone disease, pancreatitis, muscle weakness and hypercalcemic crisis^[1,3–5]^ would occur. Therefore, the fetus would suffer from neonatal death, intrauterine growth restriction, low birth weight, and hyperparathyroidism.^[4,5,14–16]^ In this case, to resolve the hypercalcemic crisis, instant CRRT was performed effectively to correct the calcium level in the patient's blood, and to attenuate the risks of complications for both mother and fetus. After the surgery, the patient's calcium dropped quickly, calcium gluconate injection, calcium carbonate and vitamin D3 tablets were administered to maintain the calcium level to avoid congenital rickets. Worsening hypercalcemia may cause acute pancreatitis during pregnancy or after delivery.^[3]^ Abdominal pain could be a sign of acute pancreatitis or threatened abortion, which will be distinguished by whether there was any decreased fetal movement or colporrhagia, and the lab test of serum amylase would provide the evidence. In this case, threatened abortion was observed 3 days after the operation. Magnesium sulfate solution was administered to prevent the abortion. The pain was relieved 1 day after the magnesium sulfate treatment, and the fetal movement was back to normal.

Both high and low serum calcium levels are risk factors for mother and baby. It is important to monitor the serum calcium level during the pregnancy to prevent maternal and fetal complications.

### 3.3 Monitoring the fetus

The fetus is exposed to maternal hypercalcemia in utero, which may inhibit fetal parathyroid function or lead to permanent hypoparathyroidism because of impaired parathyroid gland development. Some studies revealed that patients had an increased chance of miscarriage due to the parathyroid adenoma.^[6,10,17]^ In this case, the patient was suffering from threatened abortion, and magnesium sulfate was used to ensure safe gestation. Norman et al reported that patients having an average serum calcium level of 2.9 mmol/L would place them in approximately 70% of miscarriage.^[6]^ Once the pregnant women with PHPT suffered from abdominal pain and/or vaginal bleeding, it may be a signal of abortion. Meanwhile, obstetric consultation and fetal heart monitoring sounds are needed in preventing abortion.

### 3.4 Treatment

There is no standard guideline for treating PHPT during pregnancy. Till now, physicians treat the maternal PHPT empirically, and the treatment choices include a conservative approach or surgery. Maternal PHPT can be managed conservatively with oral or intravenous rehydration with or without forced diuresis,^[18,19]^ oral phosphates, a low-calcium diet and vitamin D supplementation. The role of bisphosphonates and calcimimetics as a medical therapy has not been proven safe. Bisphosphonates may cross the placenta, so their use should be restricted to life-threatening hypercalcemia due to the risk of side effects on fetal skeletal development. Calcitonin can be used during pregnancy with caution, as it does not cross the placenta. However, its use is limited because of insufficient safety data and poor effectiveness. Some studies supported that surgical removal of adenoma was the first-line treatment during the second trimester of pregnancy,^[6,13,18,20–23]^ which would minimize the risk of precipitating preterm delivery and avoid fetal exposure to anesthetic agents during organogenesis. The serum calcium level higher than 2.8 mmol/L and prior miscarriage history are also indicators to be considered for surgery.^[6,10,24,25]^ However, urgent surgery is recommended regardless of the gestation age if all therapies are performed unsuccessfully or the mother and fetus complications arise. Focused or minimally invasive parathyroidectomy (MIP) is the golden PHPT therapeutic standard in pregnancy.^[21]^ In this case, the patient has a history of abortion, serum calcium level has been higher than 3 mmol/L. In the second trimester of pregnancy, parathyroidectomy was the best choice for the patient after a multidisciplinary consult. Surgeons removed the parathyroid adenoma successfully without any complications in both mother and fetus.

### 3.5 The decision regarding surgery for a pregnant woman with primary

Hyperparathyroidism is more complex than in men or nonpregnant women. Physicians need to consider the side effects of medicines and risks of invasive therapies that may cause intrauterine growth retardation, prematurity and intrauterine fetal loss. Furthermore, there are no sample-based guidelines to assist clinicians in treating primary hyperparathyroidism during pregnancy. The decision should be made based on the severity of hypercalcemia level and symptoms.

## 4 Conclusions

PHPT during pregnancy is rare and hard to diagnose and can cause life-threatening complications for mother and fetus. In this case report, a case of PHPT observed during pregnancy is presented. It provides valuable information on early diagnosis and treatment, which would help increase clinician's awareness of PHPT during pregnancy.

## Author contributions

**Writing – original draft:** Yun Xu.

**Writing – review & editing:** Yingying Yu.
